# Texture-based block partitioning method for motion compensated frame interpolation

**DOI:** 10.1186/s40064-016-3504-7

**Published:** 2016-10-21

**Authors:** Ho Sun Jung, Myung Hoon Sunwoo

**Affiliations:** 1 Korea Testing Laboratory, 10, Chungui-ro, Jinju-si, Gyeongsangnam-do Korea; 2 School of Electronics and Computer Engineering, Ajou University, San 5, World cup-ro, Yeongtong-gu, Suwon, 16499 Korea

**Keywords:** Block matching algorithm (BMA), Distributed video coding (DVC), Frame interpolation, Frame rate up-conversion (FRUC), Motion estimation (ME)

## Abstract

This paper presents a novel motion compensated frame interpolation (MCFI) algorithm that includes texture-based wedgelet partitioning (TWP) and multiple prediction based search (MPS). TWP partitions a rectangular block into two wedge-shaped sub-blocks using the texture information, which makes a better approximation for an actual object region. Thus, detailed motions around the object boundaries can be more precisely represented than by existing MCFI algorithms. To reliably estimate the actual motion, the MPS algorithm is used in addition to TWP. MPS considers the distances between the predicted motion vectors and the candidate motion vectors, as well as the matching error. Experimental results reveal that the proposed MCFI can improve the average peak signal-to-noise ratio performance by up to 2.93 dB compared to existing MCFIs. On the average structural similarity metric, the proposed MCFI algorithm is superior to existing algorithms by a value of up to 0.0256. In addition, the proposed MCFI can reduce the computational complexity by as much as 66.9 % with respect to the sum of absolute difference compared with existing MCFIs.

## Background

Frame interpolation, a technique to upconvert the video frame rate from a lower one into a higher one, has been recognized as important since the advent of television standards (e.g., NTSC and PAL) having different frame rates (Thomas 1987; de Haan 2000). Aside from the video format conversion, frame interpolation is applicable to many video applications such as slow-motion playback and low bit-rate video coding, since it makes a video more fluid (Huynh-thu and Ghanbari 2008). Recently, it has also been used to alleviate display motion blur in hold-type displays, such as a liquid crystal display, by increasing the frame rate from 30 (or 25) Hz to 120 (or 100) Hz, or even to 240 (or 200) Hz (Someya and Sugiura 2007).

Simple approaches to frame interpolation, such as frame repetition or averaging, can produce inadequate results that exhibit motion judder or blur (de Haan 2000). To address this problem, motion compensated frame interpolation (MCFI) has been proposed (Hsu and Chien 2008; Qian and Bajic 2013; Ponla et al. 2009; Mahajan et al. 2009; Han and Woods 1997; Kim and Sunwoo 2014; Wang et al. 2010; Ha et al. 2004; Gunyel and Alatan 2010; Choi et al. 2007; Kang et al. 2008, 2010). MCFI generally has two primary steps: motion estimation (ME) and motion compensation (MC). ME calculates motion vectors (MVs) in moving images, and MC generates a new interpolated frame using the MVs.

Several MCFI algorithms have been proposed in recent years (Hsu and Chien 2008; Qian and Bajic 2013; Ponla et al. 2009; Mahajan et al. 2009; Han and Woods 1997; Kim and Sunwoo 2014; Wang et al. 2010; Ha et al. 2004; Gunyel and Alatan 2010; Choi et al. 2007; Kang et al. 2008, 2010), and they can be classified into three types with respect to ME. The first one is global MCFI (Hsu and Chien 2008; Qian and Bajic 2013; Ponla et al. 2009), which employs global ME (Chen et al. 2008) to find the global motion that has occurred between two frames. Global ME uses a set of parameters—the global MV—to describe the motion, instead of many local MVs. Global MCFI is able to handle camera motions such as scaling, rotation, and translation. However, jitter effects can occur on moving objects in global MCFI when the direction of motion of the objects is different from that of the camera (Hsu and Chien 2008; Qian and Bajic 2013; Ponla et al. 2009). Furthermore, global MCFI demands considerable computational power and large memory bandwidth (Chen et al. 2008).

**Fig. 1:**
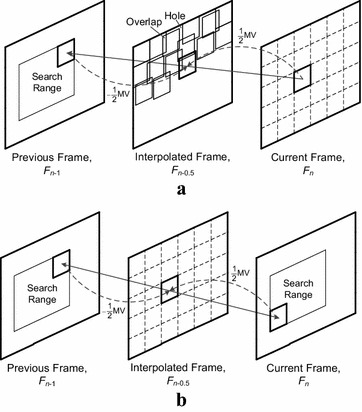
Block-wise MCFI. **a** Unilateral BMA. **b** Bilateral BMA

The second class of MCFI algorithms is pixel-wise MCFI (Mahajan et al. 2009; Tang and Au 1998), which estimates and compensates motions at pixel level. Therefore, it can interpolate the boundary regions of moving objects more precisely, but it also requires far more computational load than block-wise MCFI (Tang and Au 1998). The last one, block-wise MCFI (Han and Woods 1997; Kim and Sunwoo 2014; Wang et al. 2010; Ha et al. 2004; Gunyel and Alatan 2010; Choi et al. 2007; Kang et al. 2008, 2010) uses a block-matching algorithm (BMA). BMA is widely used for MCFI as it is simple and easy to implement, even though it introduces blocking artifacts (Tang and Au 1998). BMA divides a frame into blocks and estimates the movement of those blocks. Then, the half-magnitudes of the MVs obtained from BMA are assigned to the corresponding blocks to interpolate a new frame, as shown in Fig. 1.

Two kinds of BMA are primarily used for block-wise MCFI: unilateral BMA (Han and Woods 1997; Kim and Sunwoo 2014; Wang et al. 2010; Ha et al. 2004; Gunyel and Alatan 2010) and bilateral BMA (Choi et al. 2007; Kang et al. 2008, 2010). Figure 1a, b shows the unilateral and bilateral BMA, respectively. A unilateral BMA divides the current frame Fn into blocks, estimates the MV of each block with respect to the previous frame Fn-1, and interpolates a new frame Fn-0.5 along the MVs. Therefore, as shown in Fig. 1a, the unilateral BMA results in holes and overlaps.

A bilateral BMA, on the other hand, can avoid hole and overlap problems. It divides the frame to be interpolated into blocks and estimates the MV of each block using the temporal symmetry between the current and previous frames. Then, the bilateral BMA interpolates a new frame along the MVs. Thus, it avoids holes and overlaps, as shown in Fig. 1b. However, the bilateral BMA exhibits lower peak signal-to-noise ratio (PSNR) performance than a unilateral BMA because a BMA based on an unknown block in the intermediate frame has limited prediction accuracy (Choi et al. 2007; Kang et al. 2008, 2010). If there is spurious temporal symmetry in a patterned (or unicolor) background, or from similar (or identical) objects in the previous and current frames, the bilateral BMA can give incorrect MVs. This phenomenon limits its prediction accuracy and degrades image quality.

The prediction accuracy and speed of ME are key issues to consider in the construction of reliable interpolated frames. Unlike video compression, ME in block-wise MCFI finds true motion trajectory rather than one with the minimum matching error (e.g., the sum of squared differences between the two blocks in the current and previous frames) (Thomas 1987). Several approaches for more accurate ME have been proposed in recent works (Wang et al. 2010; Ha et al. 2004; Gunyel and Alatan 2010; Choi et al. 2007; Kang et al. 2008, 2010; Wang et al. 2010). The algorithms in Wang et al. (2010), Gunyel and Alatan (2010), Choi et al. (2007) iterate the ME process to refine a MV field, while the algorithms in Ha et al. **(** 2004), Kang et al. (2008, 2010) and Wang et al. (2010) generate multiple MV fields and pick one reliable field. As a result, these algorithms (Wang et al. 2010; Ha et al. 2004; Gunyel and Alatan 2010; Choi et al. 2007; Kang et al. 2008, 2010; Wang et al. 2010) may achieve relatively good performance but also lead to huge computational requirements, since ME is a highly computation-intensive task.

**Fig. 2:**
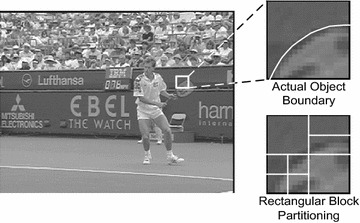
Example of rectangular block partitioning

Block size selection, meanwhile, is critical to any block-based ME. A larger block size is advantageous to more stable motion, since it has more texture information. However, a larger block size is worse at representing the motion characteristics around moving object boundaries. Hence, finding an optimal block size is an ill-posed problem (Gunyel and Alatan 2010; Choi et al. 2007). Several MCFI algorithms using variable block sizes have been proposed to address this problem (Gunyel and Alatan 2010; Choi et al. 2007). However, the arbitrary shape of an actual object region with a uniform MV field cannot be precisely represented by rectangular blocks, as shown in Fig. 2. Hence, pixels in a rectangular block may belong to different objects that have different actual motion characteristics, such as the tennis racket and the fence in Fig. 2.

**Fig. 3:**
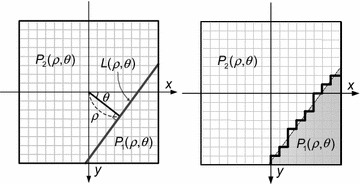
Partition line and two wedge-shaped parts in WBP method

In video coding, wedgelet block partitioning (WBP) (Kondo and Sasai 2005; Hung et al. 2006) is used to better partition blocks. In the WBP method, blocks are partitioned by a straight line into two wedge-shaped sub-blocks, P1 and P2. The straight partition line L(ρ, θ) is defined by two parameters: the radius ρ denoting the distance between the partition line and the center of the block, and the angle θ between the partition line and the y-axis, as shown in Fig. 3. A detailed explanation of Fig. 3 is offered in “Texture-based wedgelet partitioning” section. Since wedge-shaped sub-blocks can be matched more closely to the boundary regions of moving objects, the prediction accuracy of block-based ME can be improved by using WBP (Kondo and Sasai 2005; Hung et al. 2006).

In summary, the prediction accuracy of ME directly affects the interpolation performance of MCFI, and existing algorithms (Han and Woods 1997; Kim and Sunwoo 2014; Wang et al. 2010; Ha et al. 2004; Gunyel and Alatan 2010; Choi et al. 2007; Kang et al. 2008, 2010) are either too complicated to implement or cannot provide reasonable performance with low computational complexity. Furthermore, existing algorithms (Han and Woods 1997; Kim and Sunwoo 2014; Wang et al. 2010; Ha et al. 2004; Gunyel and Alatan 2010; Choi et al. 2007; Kang et al. 2008, 2010) that use fixed rectangular blocks cannot estimate precise motion near object boundaries.

This paper proposes an efficient MCFI algorithm that includes texture-based wedgelet partitioning (TWP) and multiple prediction based search (MPS). TWP partitions a block into two wedge-shaped sub-blocks using texture information. The variable-sized and shaped blocks of TWP, which are close to the actual object region, can precisely represent detailed motions around the object boundaries. In addition, MPS that considers the correlation among neighboring MVs can find accurate motion trajectories of blocks of variable size and shape with relatively low computational complexity. To obtain reliable MVs, MPS uses a cost function that takes into account the distances between predicted vectors and candidate vectors as well as the matching error. Consequently, the quality of the interpolated frame can be noticeably improved using TWP and MPS.

The rest of this paper is organized as follows. “Proposed MCFI algorithm” section describes the proposed MCFI algorithm consisting of TWP and MPS. “Experimental results” section presents the experimental results and various performance comparisons. Finally, we offer our conclusions in “Conclusion” section.

## Proposed MCFI algorithm

**Fig. 4:**
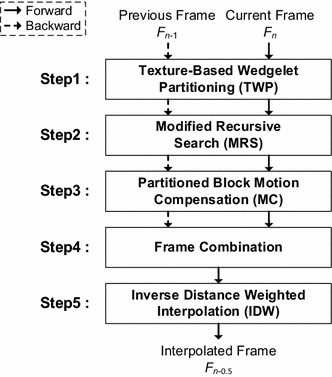
Flowchart of the proposed MCFI

This section introduces a novel MCFI algorithm that interpolates a new frame *F*_*n*−∆_ using the unilateral BMA between adjacent original frames, *F*_*n*−1_ and *F*_*n*_. For the sake of concreteness, ∆ here is set to 0.5, but any value of ∆ between 0 and 1 can be used by appropriately scaling the MVs. Figure 4 depicts a flowchart of the proposed MCFI algorithm.

To construct a new frame *F*_*n*−0.5_, the proposed algorithm first partitions two adjacent original frames, *F*_*n*−1_ and *F*_*n*_, into wedge-shaped sub-blocks according to TWP. Then, MPS estimates two MV fields, forward and backward, which are associated with each partitioned block in two adjacent frames, *F*_*n*−1_ and *F*_*n*_. The forward MVs can be obtained as shown in Fig. 1a, whereas the backward MVs can be obtained by finding the block in the current frame that is most similar to that in the previous frame. Following MPS, MC—shown as Step3 of the flowchart—is performed twice using both the forward and backward MV fields to construct two intermediate frames. These two frames are then combined to reduce holes and blocking artifacts (Kim and Sunwoo 2014). Since holes remain in the combined frame, the proposed algorithm employs inverse distance weighting (IDW) (Lu and Wong 2008) to fill these holes at low computational complexity. Details of the functional blocks in Fig. 4 are presented in the following subsections.

### Texture-based wedgelet partitioning

As shown in Fig. 3, the block partitioning is determined by two partitioning parameters, *ρ* and *θ*, in WBP. Based on the partition line *L*(*ρ*, *θ*), the partitioning function for pixel coordinates (*x*, *y*) can be expressed as

1 $$f(x,y) = x\cos \theta + y\sin \theta - \rho$$

with the origin of the coordinate system at the center of the block. For a given partition line, the pixels in the block are partitioned into two wedge-shaped sub-blocks, *P*_1_(*ρ*, *θ*) and *P*_2_(*ρ*, *θ*), as follows:

2 $$P(x,y) = \left\{ {\begin{array}{*{20}c} {P_{1} (\rho ,\theta ) \, ,\quad {\text{if}}\; \, f(x,y) \ge 0} \\ {P_{2} (\rho ,\theta ) \, ,\quad {\text{if }}\;f(x,y) < 0} \\ \end{array} } \right..$$

After the block is partitioned according to the given candidate partition line, the existing WBP method (Kondo and Sasai 2005; Hung et al. 2006) for video coding performs ME on each partitioned sub-block. Then, the partition line that has the minimum rate-distortion cost is selected as the best one. However, MCFI does not need to take the bit-rate cost into account. Moreover, the partition line that has the minimum matching error does not always correspond with the actual object boundary. Minimizing matching error can improve the rate-distortion performance in video coding, but reliably approximating the actual object boundary is more important in MCFI to achieve high performance (Choi et al. 2007; Kondo and Sasai 2005). In addition, an exhaustive computation overhead is involved in performing ME on the full set of candidate partition lines.

To obtain the best partition line representing the actual object boundary and to reduce the search complexity, the proposed TWP method uses texture information. Regions belonging to different objects with different MVs are very likely to have different textures. Therefore, if a block contains two parts with different MVs, a good partition line should partition the block into two sub-blocks with the largest texture difference. The texture difference can be estimated using the mean and variance values of the two sub-blocks. Consequently, the best partition line should maximize the difference between the mean values of the two sub-blocks and minimize the sum of the two variance values.

Let *μ* and *v* denote the mean and variance values of each wedgelet sub-block, respectively. Then, the criterion for selecting the best partition line *L*(*ρ*_*B*_, *θ*_*B*_) can be formulated as

3 $$(\rho_{B} ,\theta_{B} ) = \mathop {\arg \hbox{max} }\limits_{(\rho ,\theta ) \in PS} \left( {\frac{{\left| {\mu (P_{1} (\rho ,\theta )) - \mu (P_{2} (\rho ,\theta ))} \right| + \alpha_{1} }}{{v(P_{1} (\rho ,\theta )) + v(P_{2} (\rho ,\theta )) + \alpha_{2} }}} \right),$$

where *PS* denotes the candidate set of partitioning parameters (*ρ*, *θ*). Two variables *α*_1_ and *α*_2_ are included to stabilize the division. By (3), we can determine the optimal partition line that maximizes the texture difference. Since calculating variance requires the sum of squared differences (SSD) operation, TWP uses the sum of absolute differences (SAD) instead of SSD to reduce the computational complexity. Experimental results show that using SAD instead of SSD does not affect partitioning results.

For a 16 × 16 block, the search ranges for *ρ* and *θ* are [0, 8) and [0, 2*π*), respectively. Thus, there are (8/Δ*ρ*) × (2*π*/Δ*θ*) candidate partition lines for a 16 × 16 block, where Δ*ρ* and Δ*θ* are the associated search step sizes of *ρ* and *θ*, respectively. Although the small search step size of partitioning parameters leads to good interpolation performance, there is a huge computational cost in examining a large number of candidate partition lines. Since both PSNR performance and computational cost are reasonably good at Δ*ρ* = 1 and Δ*θ* = *π*/8 for various experiments using TWP, these search step sizes are used in this work. Moreover, the search range of *ρ* is restricted to [0, 5) in the proposed block partitioning method because the larger value of *ρ* makes one of two wedgelet sub-blocks smaller, and that sub-block may lose its texture characteristic.

**Fig. 5:**
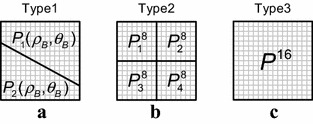
Three types of block partitioning. **a** Type1 (wedgelet). **b** Type2 (8 × 8). **c** Type3 (16 × 16)

**Fig. 6:**
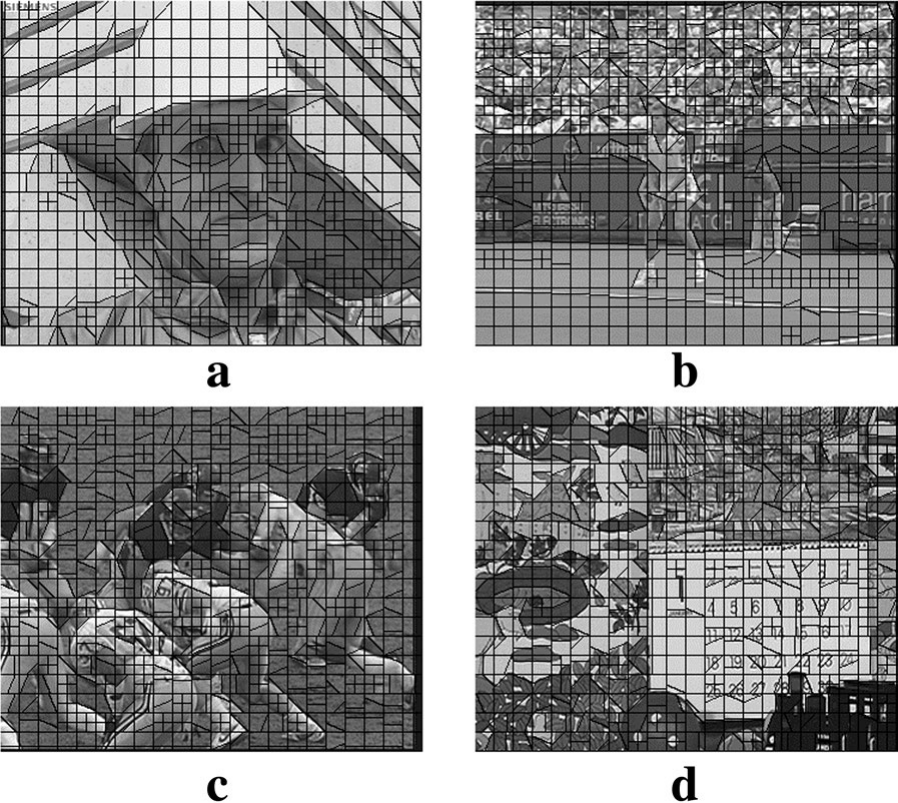
Block partitioning results using TWP. **a** Foreman. **b** Stefan. **c** Football. **d** Mobile

TWP also uses a 16 × 16 block and four 8 × 8 sub-blocks in lieu of two wedgelet sub-blocks. Figure 5 illustrates the three types of block partitioning: wedgelet (Type1), 8 × 8 (Type2) and 16 × 16 (Type3). To determine the best partitioning among these types, the mean and variance values of a 16 × 16 block (*P* ^16^) and four 8 × 8 sub-blocks (*P*_1_^8^, *P*_2_^8^, *P*_3_^8^ and *P*_4_^8^) are compared with those of two wedgelet sub-blocks in a similar manner as (3). For Type2, the difference between the two wedgelet variance values in (3) is compared with {*ν*(*P*_1_^8^) + *ν*(*P*_2_^8^) + *ν*(*P*_3_^8^) + *ν*(*P*_4_^8^)}/2, and the sum of the two wedgelet mean values in (3) is compared with |*μ*(*P*_1_^8^) + *μ*(*P*_2_^8^) + *μ*(*P*_3_^8^) + *μ*(*P*_4_^8^) − 4*μ*(*P* ^16^)|/2. In case of Type3, the sum of the two wedgelet variances is only compared with 1.5*ν*(*P* ^16^) in this paper, since *P* ^16^ only has one region. Figure 6 shows the block partitioning results using TWP. Note that the partitioning results correspond closely with the boundaries of moving objects.

### Multiple prediction based search

**Fig. 7:**
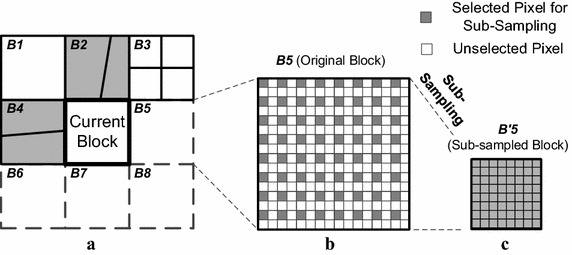
Example of neighboring blocks for the predicted MVs and formation of the sub-sampled block. **a** Neighboring blocks. **b** Original block B5. **c** Sub-sampled block B*′*5

Following the TWP, ME is carried out for each partitioned sub-block using the proposed MPS—Step2 in Fig. 4. MPS uses the MVs of neighboring blocks to predict the MV of the current block. Figure 7a shows an example of the neighboring blocks. The neighboring blocks usually depict the same moving object as the current block. Hence, the estimation results around the current block can be good predictors of the current block motion.

To take advantage of the correlation between the current and neighboring blocks, the algorithm in Kim and Sunwoo (2014) uses the MVs of *B1*, *B2* and *B4* blocks as the predicted MVs. However, the MVs of *B5* and *B7* are not used for the prediction in Kim and Sunwoo (2014) because those blocks do not have MVs yet. However, it is possible that the current block represents an actual moving object that is depicted only in *B5* and *B7.* This would mean that the algorithm in Kim and Sunwoo (2014) cannot predict an accurate MV. To address this problem, the proposed MPS uses the MV of *B5* as shown in Fig. 7a. In order to reduce the computation complexity, the sub-sampled block *B′5* is used, which is generated from *B5*. Hence, the MV of the sub-sampled block *B’5* is used as the predicted MV, because the predicted MV from *B′5* may indicate the same object in the current block. To reduce the computation complexity, the MVs of *B6, B7 and B8* are excluded, and thus, MPS can greatly reduce the computation cost.

MPS uses the MVs from *B2*, *B4* and sub-sampled block *B′5* as the predicted MVs. The sub-sampled block *B′5* is formed from the original block *B5* as shown in Fig. 7b, c, and ME is performed on the sub-sampled block to obtain the predicted MV.

**Fig. 8:**
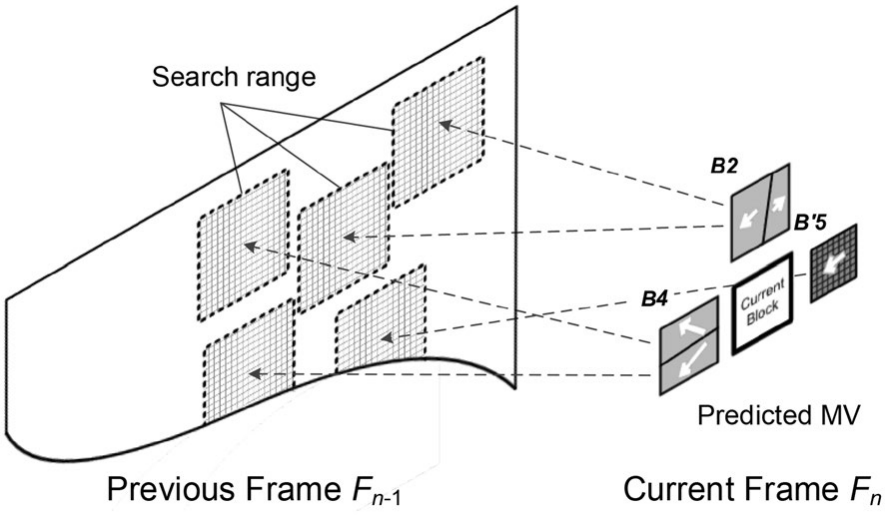
Example of the search range for the current block

The five predicted MVs from the shaded blocks in Fig. 7 (i.e., *B2, B4* and *B′5*) are used as the center of the search range as shown in Fig. 8. MPS selects the MV of the current block only among a very limited number of candidate vectors arranged around the centers of search points. To consider the predicted MVs more effectively, we also propose the following cost function to select the MV of the current block:

4 $${\text{MV}} = \mathop {\arg \hbox{min} }\limits_{C \in CS} \left\{ {e(C) \times \left( {1 + \lambda \sum\limits_{i = 1}^{5} {d_{i} } } \right)} \right\},$$

where *CS* is the set to which the candidate vector belongs, and *e*(*C*) stands for the matching error corresponding to *C*. The matching error is calculated using SAD between the two blocks (or sub-blocks), the current block and the candidate block in the previous frame corresponding to *C*. The distance between *C* and one of the predicted MVs is denoted by *d*_*i*_, and the sum of the distances is weighted with the matching error by *λ*. In this work, *λ* has been empirically set to 0.025.

The predicted MVs from the neighboring blocks, *B2* and *B4*, are different according to their block types shown in Fig. 5. In the case of Type1, all the MVs of the two wedgelet sub-blocks are used as the predicted MVs because each partitioned sub-block usually belongs to a different object, and the current block may belong to either of the objects. Type3, on the other hand, has only one MV, which is used twice for the above cost function as the predicted MV. In Type2, only two MVs, from *P*_2_^8^ and *P*_3_^8^, of the four are used as the predicted MVs. Using the proposed cost function, MPS can effectively consider the correlation of the candidate vectors with the predicted MVs as well as the matching error.

### Partitioned block interpolation

This subsection details the remainder of the MCFI procedure, including Step3, Step4 and Step5 as shown in Fig. 4. The forward and backward MV fields obtained from MPS are used to construct the forward and backward intermediate frames, *F* ^*f*^ and *F* ^*b*^, respectively. The forward intermediate frame *F* ^*f*^ is given by

5 $$F^{f} \left( {x + \frac{{m_{x} }}{2},y + \frac{{m_{y} }}{2}} \right) = \frac{1}{2}\left\{ {F_{n - 1} (x + m_{x} ,y + m_{y} ) + F_{n} (x,y)} \right\},$$

where *m*_*x*_ and *m*_*y*_ denote the *x* and *y* components of the forward MV associated with the pixel coordinates (*x*, *y*). The backward intermediate frame is also constructed in a similar manner. In this step, the quality of the intermediate frames may be enhanced by using gradient values instead of pixel intensities (Mahajan et al. 2009). Constructing the intermediate frames in the gradient domain helps to preserve the edges better and hence reduce blurring. However, it can significantly increase the computational complexity, and thus this work does not use the gradient method.

As described earlier, overlaps and holes are generated according to the MV trajectory in the unilateral BMA (Han and Woods 1997; Kim and Sunwoo 2014; Wang et al. 2010; Ha et al. 2004; Gunyel and Alatan 2010). In the unilateral BMA, even though a region does not contain any complicated information such as texture or edges, overlaps and holes may be appeared in the motion compensated frames (Han and Woods 1997; Kim and Sunwoo 2014; Wang et al. 2010; Ha et al. 2004; Gunyel and Alatan 2010). Since the proposed MCFI also uses the unilateral BMA, it also shows overlaps and holes. Overlaps can be resolved by averaging overlapped pixels, but blocking artifacts may appear as a result. Therefore, to reduce blocking artifacts and holes, a frame combination (Kim and Sunwoo 2014) is necessary. The proposed MCFI combines the two intermediate frames *F* ^*f*^ and *F* ^*b*^ into one frame *F*_*n*−0.5_ as follows:

6 $$F_{n - 0.5} (x,y) = \left\{ {\begin{array}{*{20}l} {\frac{{F^{f} (x,y) + F^{b} (x,y)}}{2}} \hfill & \quad {{\text{if }}F^{f} (x,y) \ne {\text{Hole and }}F^{b} (x,y) \ne {\text{Hole}}} \hfill \\ {F^{f} (x,y)} \hfill & \quad {{\text{if }}F^{f} (x,y) \ne {\text{Hole and }}F^{b} (x,y) = {\text{Hole}}} \hfill \\ {F^{b} (x,y)} \hfill & \quad {{\text{if }}F^{f} (x,y) = {\text{Hole and }}F^{b} (x,y) \ne {\text{Hole}}} \hfill \\ {\text{Hole}} \hfill & \quad{\text{otherwise}} \hfill \\ \end{array} } \right..$$

If neither *F* ^*f*^(*x*, *y*) nor *F* ^*b*^(*x*, *y*) is a hole, the average of the two values is taken as the final pixel value of *F*_*n*−0.5_(*x*, *y*). On the other hand, if one of either *F* ^*f*^(*x*, *y*) or *F* ^*b*^(*x*, *y*) is a hole, the frame combination uses the other, which is not a hole, as the final pixel value. Otherwise, if both *F* ^*f*^(*x*, *y*) and *F* ^*b*^(*x*, *y*) are holes, the pixel remains a hole.

**Fig. 9:**
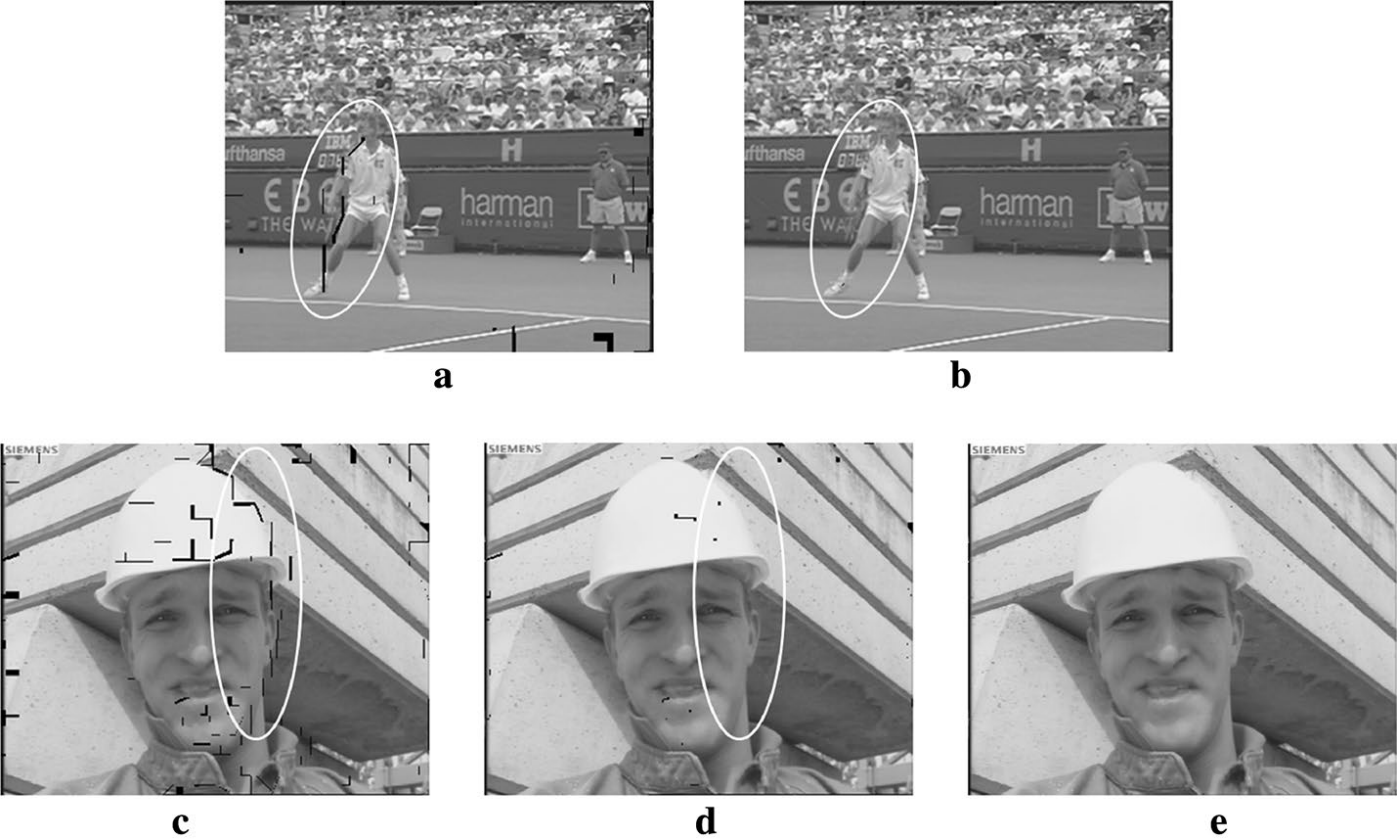
Comparisons of holes before and after the frame combination. **a** Forward intermediate frame of Stefan. **b** Combined frame of Stefan. **c** Forward intermediate frame of Foreman. **d** Combined frame of Foreman. **e** Interpolated frame based on (7)

During the frame combination, holes are reduced and the image quality improves (Kim and Sunwoo 2014). Figure 9 compares holes in images before and after frame combination. The irregular black spots highlighted by white circles represent holes. Figure 9a, c shows the holes only in the forward intermediate frame, whereas Fig. 9b, d shows those in the combined frame. These figures clearly show that the frame combination considerably reduces the number of holes. While many holes are apparent near the moving object boundaries in Fig. 9a, c, holes in Fig. 9b, d are substantially reduced.

Since the combined frame may contain regions with holes even after the frame combination, the remaining holes should be interpolated using adjacent pixel information. To interpolate the remained holes, the proposed MCFI employs IDW (Lu and Wong 2008). IDW, one of the most frequently used deterministic models in spatial interpolation, is a straightforward method that is not computationally intensive (Lu and Wong 2008). The hole located at (*x*_0_, *y*_0_) is filled using the following equation:

7 $$F_{n - 0.5} (x_{0} ,y_{0} ) = \sum\limits_{j = 1}^{4} {\frac{{d_{j}^{ - 1} F_{n - 0.5} (x_{j} ,y_{j} )}}{{\sum\nolimits_{j}^{4} {d_{j}^{ - 1} } }}} ,$$

where (*x*_*j*_, *y*_*j*_) is the neighboring pixel location, and *d*_*j*_^−1^ denotes the inverse of the distance between the hole and the neighboring pixel. The four neighboring pixels used for hole interpolation are the closest to the hole in both the horizontal and vertical directions. However, IDW may show blur effect in object boundaries as the limitation.

## Experimental results

In this section, we describe various experiments conducted for performance comparisons between the proposed MCFI algorithm and existing algorithms (Han and Woods 1997; Kim and Sunwoo 2014; Choi et al. 2007; Kang et al. 2010). The search range in each experiment was set to ± 16 pixels for both horizontal and vertical directions, but only the proposed MCFI used the search range of ± 4 because of MPS. All test sequences used for the experiments are in the standard CIF (352 × 288), 720p (1280 × 720) and 1080p (1920 × 1080) formats. To quantitatively measure the quality of the interpolated frames, 50 odd frames were removed from 30 fps test sequences and new 50 odd frames were reconstructed using 51 even frames. Then, PSNR performances of the reconstructed frames were compared with the original odd frames. Since most of the experimental literature on MCFI (Kim and Sunwoo 2014; Wang et al. 2010; Ha et al. 2004; Choi et al. 2007) uses 50 frames to evaluate PSNR performance, we follow suit.

Table 1 lists PSNR comparisons between various MCFI algorithms (Han and Woods 1997; Kim and Sunwoo 2014; Choi et al. 2007; Kang et al. 2010) and our proposed method. Both OB-MCI (Han and Woods 1997) and PMVS (Kim and Sunwoo 2014) use the unilateral BMA, while VS-BMC (Choi et al. 2007) and Dual ME (Kang et al. 2010) use the bilateral BMA. OB-MCI (Han and Woods 1997) is a typical MCFI that considers only the matching error to obtain MV fields. In addition, OB-MCI fills the holes due to the unilateral BMA using pixel values from the previous frame or the current frame. In contrast, PMVS (Kim and Sunwoo 2014) considers the correlation among neighboring MVs for MV smoothing and analyzes the orientation of the edges to cover holes and overlaps. To overcome the limited prediction accuracy of the bilateral BMA, Dual ME (Kang et al. 2010) uses the unilateral ME with enlarged blocks as well as the bilateral ME, and VS-BMC (Choi et al. 2007) takes into account the side match distortion (SMD) for ME. Furthermore, VS-BMC uses variable-sized rectangular blocks to estimate detailed motions.

**Table 1:** PSNR comparison of different MCFI algorithms

Sequence		OB-MCI (Han and Woods 1997) (dB)	PMVS (Kim and Sunwoo 2014) (dB)	VS-BMC (Choi et al. 2007) (dB)	Dual ME (Kang et al. 2010) (dB)	Proposed (dB)
*Foreman*	CIF	31.18	32.71	32.81	32.87	33.58
*Akiyo*		41.47	45.11	44.87	45.02	45.84
*Football*		20.83	21.71	21.32	20.75	24.09
*Coastguard*		27.76	30.75	28.28	29.37	31.66
*Flower*		26.56	27.93	25.83	28.85	28.42
*Highway*		30.92	31.53	31.27	31.38	32.69
*Mobile*		22.62	26.18	22.54	24.22	28.02
*News*		34.50	36.25	34.86	35.47	36.67
*Hall*		34.71	34.42	34.75	35.25	35.33
*Stefan*		24.11	26.40	23.15	25.84	28.68
*Silent*		33.81	34.80	34.17	34.52	34.94
*Container*		37.89	38.19	38.65	38.41	38.77
*Parkrun*	720p	23.38	26.09	21.70	22.55	27.52
*Shields*		31.17	34.53	32.02	32.84	35.89
*Stockholm*		29.12	33.04	30.38	31.02	33.65
*Vidyo1*		42.10	43.84	43.15	44.53	45.52
*FourPeople*		38.18	36.68	38.57	39.54	40.56
*Johnny*		38.25	32.24	40.70	41.12	41.35
*Bluesky*	1080p	23.94	26.61	24.50	24.84	29.29
*Sunflower*		30.82	31.57	30.02	30.21	31.56
*Kimono1*		30.22	31.28	30.01	32.65	32.62
*ParkScene*		29.85	31.36	28.68	31.13	31.17
Average		31.06	32.42	31.47	32.38	33.99
Average gain		2.93	1.57	2.53	1.61	

For most of the test sequences, the proposed MCFI outperforms the existing MCFI algorithms (Han and Woods 1997; Kim and Sunwoo 2014; Choi et al. 2007; Kang et al. 2010) in terms of PSNR. As shown in Table 1, the PSNR for our proposed MCFI is on average 2.93, 1.57, 2.53 and 1.61 dB greater than OB-MCI, PMVS, VS-BMC and Dual ME, respectively. In particular in the *Football* and *Stefan* sequences, the proposed MCFI improves PSNR at least by 2.28 dB and at most by 5.53 dB in comparison with existing MCFI algorithms (Han and Woods 1997; Kim and Sunwoo 2014; Choi et al. 2007; Kang et al. 2010). The sequences, such as Highway, Parkrun, Hall, Football, Stefan, and Kimono1 containing occlusion, rapid motion, and scene transition, are used. The proposed MCFI achieves 2.79, 1.58, 3.12 and 2.09 dB average PSNR gains compared to Han and Woods (1997), Kim and Sunwoo (2014), Choi et al. (2007) and Kang et al. (2010), respectively for the above sequences. Since detailed motions around object boundaries can be more effectively represented using TWP, notable PSNR improvements can be achieved for the sequences containing multiple objects with complicated motions. The proposed algorithm performs better than existing algorithms even for the 720p and 1080p sequences, as shown in Table 1.

As PSNR is not always consistent with the quality perceived by the human visual system (HVS), the quality of the interpolated frames was also evaluated by structural similarity (SSIM) (Wang et al. 2004) measures designed to improve upon traditional methods such as PSNR and mean squared error (Wang et al. 2004).

The SSIM comparisons are presented in Table 2. For most of the test sequences, the proposed MCFI achieves the best SSIM results among MCFI algorithms (Han and Woods 1997; Kim and Sunwoo 2014; Choi et al. 2007; Kang et al. 2010). As with PSNR, for the sequences containing complicated motions—*Football* and *Mobile,* to wit—the proposed MCFI provides at least 0.0174 and at most 0.0996 better SSIM than existing algorithms (Han and Woods 1997; Kim and Sunwoo 2014; Choi et al. 2007; Kang et al. 2010). Moreover, SSIM gains achieved by the proposed method are 0.0356, 0.0141, 0.0523 and 0.0156 compared to Han and Woods (1997), Kim and Sunwoo (2014), Choi et al. (2007) and Kang et al. (2010), respectively in the sequences containing occlusion, rapid motion or scene transition. In all the sequences, the average SSIM gains achieved by the proposed MCFI are 0.0305, 0.0113, 0.0385 and 0.0132 compared with OB-MCI, PMVS, VS-BMC and Dual ME, respectively. These gains result from the prediction accuracy of the proposed MPS with TWP.

**Table 2:** SSIM comparison of different MCFI algorithms

Sequence		OB-MCI (Han and Woods 1997)	PMVS (Kim and Sunwoo 2014)	VS-BMC (Choi et al. 2007)	Dual ME (Kang et al. 2010)	Proposed
*Foreman*	CIF	0.9659	0.9725	0.9724	0.9739	0.9741
*Akiyo*		0.9953	0.9968	0.9964	0.9968	0.9975
*Football*		0.7439	0.7887	0.7547	0.7944	0.8118
*Coastguard*		0.9104	0.9514	0.9537	0.9584	0.9704
*Flower*		0.9502	0.9519	0.9529	0.9647	0.9649
*Highway*		0.9770	0.9709	0.9772	0.9690	0.9789
*Mobile*		0.8591	0.9278	0.8850	0.9043	0.9587
*News*		0.9876	0.9913	0.9891	0.9899	0.9918
*Hall*		0.9895	0.9808	0.9896	0.9801	0.9920
*Stefan*		0.9075	0.9385	0.9101	0.9368	0.9552
*Silent*		0.9821	0.9881	0.9734	0.9730	0.9896
*Container*		0.9918	0.9947	0.9971	0.9948	0.9953
*Parkrun*	720p	0.8802	0.9354	0.7685	0.9174	0.9554
*Shields*		0.9754	0.9865	0.9323	0.9883	0.9900
*Stockholm*		0.9550	0.9796	0.9250	0.9821	0.9842
*Vidyo1*		0.9963	0.9974	0.9969	0.9975	0.9978
*FourPeople*		0.9881	0.9891	0.9891	0.9902	0.9914
*Johnny*		0.9839	0.9828	0.9897	0.9903	0.9907
*Bluesky*	1080p	0.8901	0.9170	0.8376	0.9142	0.9511
*Sunflower*		0.9541	0.9688	0.9639	0.9711	0.9718
*Kimono1*		0.9442	0.9571	0.9423	0.9644	0.9626
*ParkScene*		0.9536	0.9694	0.9509	0.9662	0.9683
Average		0.9446	0.96075	0.9385	0.9599	0.9702
Average gain		0.0256	0.0094	0.0316	0.0103	–

Table 3 compares the computational complexities of the MCFI algorithms (Han and Woods 1997; Kim and Sunwoo 2014; Choi et al. 2007; Kang et al. 2010). The number of SAD operations required for processing a block of size *B* is evaluated to compare the computational complexities of the MCFI algorithms because most of the computational cost in MCFI comes from SAD. In Table 3, *L* and *S* denote the search range of the proposed MCFI and the existing MCFI, respectively, and *N* is the iteration number of VS-BMC. For all the computational complexity evaluations, *B* and *S* were set to 16, and *L* was set to 4.

**Table 3:** Computational complexity evaluations based on SAD operations

MCFI	The number of SAD operations per block
OB-MCI (Han and Woods 1997)	*B* ^2^ × (2*S* + 1)^2^ = 278,784
PMVS (Kim and Sunwoo 2014)	2{*B* ^2^ × (2*S* + 1)^2^} + 4*B* ^2^ = 558,592
VS-BMC (Choi et al. 2007)	[{*B* ^2^ × (2*S* + 1)^2^} + {4*B* × (2*S* + 1)^2^}] × *N* = 348,480 × *N*
Dual ME (Kang et al. 2010)	2{(1.5*B*)^2^ × (2*S* + 1)^2^} − {(1.5*B*)^4^ − 24(1.5*B*)^2^ + 144} = 936,432
Proposed	2[{*B* ^2^ × 5(2*L* + 1)^2^} + {(*B* / 2)^2^ × (*S* + 1)^2^} + {*B* ^2^ × (8 / Δ*ρ*) × (2*π* / Δ*θ*)}] = 309,888

The proposed MCFI and PMVS (Kim and Sunwoo 2014) use the forward and backward ME. Besides, the proposed MCFI requires the additional computation {*B* ^2^ × (8/Δ*ρ*) × (2*π*/Δ*θ*)}, since the search for (*ρ*, *θ*) in the block partitioning also requires SAD operations. Consequently, the proposed MCFI has about 11.2 % higher computational complexity than OB-MCI (Han and Woods 1997). However, the proposed MCFI provides an average of 3.05 dB better PSNR performance than OB-MCI, as shown in Table 1. On the other hand, the second term in PMVS, 4*B* ^2^, represents the computational cost of hole interpolation, which is required to determine the best direction for hole interpolation. Compared to PMVS, the proposed MCFI has about 44.5 % lower computational complexity.

To determine the block size, VS-BMC (Choi et al. 2007) should carry out the entire process iteratively, and the iteration number *N* is at least two and at most three. In addition, VS-BMC requires the additional computation {4*B* × (2*S* + 1)^2^}, to calculate SMD. Thus, the computational complexity of the proposed MCFI is at least 55.5 % lower than VS-BMC. As mentioned above, Dual ME (Kang et al. 2010) uses both bilateral and unilateral ME with the enlarged block size 1.5*B*. Hence, Dual ME has about three times as many computations as the proposed MCFI.

The computational complexity of full-high definition (FHD) can be obtained by multiplying the number of SAD computations and the number of 16 × 16 blocks. A FHD frame has 8160 blocks. Based on Table 3, the proposed method shows more computational complexity compared to OB-MCI (Han and Woods 1997) about 254 million SAD operations [=(309,888 − 278,784) SAD operations/block × 8160 blocks]. However, the proposed method provides an average of 3.72 dB better PSNR performance than OB-MCI (Han and Woods 1997). Compared to PMVC (Kim and Sunwoo 2014), the proposed method requires about 2029 million less SAD operations [=(558,592 − 309,888) SAD operations/block × 8160 blocks]. Moreover, compared to VS-BMC (Choi et al. 2007), the proposed algorithm can reduce 3159 million SAD operations [=(696,960 − 309,888) SAD operations/block × 8160 blocks]. In addition, the proposed method has about 5113 million less SAD operations [=(936,432 − 309,888) SAD operations/block x 8160 blocks] than Dual ME (Kang et al. 2010).

The execution time comparisons among the proposed method and the others are presented in Table 4. The execution time of the proposed MCFI is 554.61 s in average, which is 257.1 and 165.75 s faster than Choi et al. (2007) and Kang et al. (2010), respectively. The algorithms in Han and Woods (1997) and Kim and Sunwoo (2014) can reduce the execution times, 247.52 and 240.37 s, compared with the proposed MCFI. However, PSNRs of the algorithms in Han and Woods (1997) and Kim and Sunwoo (2014) show 2.93, 1.57 dB lower than the proposed MCFI.

**Table 4:** Execution times for the algorithms

Sequence		OB-MCI (Han and Woods 1997) (s)	PMVS (Kim and Sunwoo 2014) (s)	VS-BMC (Choi et al. 2007) (s)	Dual ME (Kang et al. 2010) (s)	Proposed (s)
*Foreman*	CIF	46.54	47.01	120.34	108.17	86.06
*Akiyo*		46.81	47.48	120.14	107.27	86.90
*Football*		46.59	47.04	120.28	107.67	82.74
*Coastguard*		46.77	47.11	120.39	107.48	85.68
*Flower*		46.59	46.82	120.59	107.37	85.10
*Highway*		46.60	47.20	120.29	107.56	84.34
*Mobile*		45.38	47.09	120.41	107.79	86.83
*News*		45.74	46.77	120.87	107.85	84.48
*Hall*		46.56	47.20	120.81	107.4	84.14
*Stefan*		45.53	46.78	120.52	107.68	82.93
*Silent*		45.47	46.76	120.46	108.56	84.50
*Container*		46.68	46.81	121.58	107.46	85.80
*Parkrun*	720p	414.00	427.00	1096.67	956.25	750.40
*Shields*		414.53	426.20	1096.07	961.77	750.22
*Stockholm*		413.64	426.44	1097.65	958.58	761.72
*Vidyo1*		400.88	417.34	1089.53	955.72	745.95
*Fourpeople*		412.17	418	1090.26	960.76	749.07
*Johnny*		409.82	418.44	1088.14	954.53	757.68
*Bluesky*	1080p	954.30	966.25	2463.30	2194.49	1697.86
*Kimono1*		922.19	942.96	2457.10	2177.59	1644.35
*ParkScene*		923.37	943.95	2452.35	2186.98	1676.42
*Sunflower*		935.67	962.61	2479.81	2249.00	1648.18
Average		307.08	314.24	811.71	720.36	554.61
Average gain		247.52	240.37	−257.10	−165.75	–

In FHD sequences, the algorithms in Han and Woods (1997) and Kim and Sunwoo (2014) are 732.82 and 712.76 s (seconds) faster than the proposed MCFI. However, the proposed MCFI provides 2.45 and 0.96 dB better PSNR and 0.028 and 0.0104 more SSIM than Han and Woods (1997) and Kim and Sunwoo (2014), respectively. In addition, the proposed MCFI can reduce about 796.44 and 535.31 s compared to Choi et al. (2007) and Kang et al. (2010), respectively. Consequently, Table 1, 2, 3 and 4 show that the proposed MCFI is the most efficient algorithm in terms of both the interpolation performance and the computational complexity among rival MCFI algorithms (Han and Woods 1997; Kim and Sunwoo 2014; Choi et al. 2007; Kang et al. 2010).

## Conclusion

This paper proposed an efficient MCFI algorithm consisting of two ideas: TWP and MPS. TWP can effectively represent detailed motions around object boundaries using variable-sized and variable-shaped blocks. In addition, MPS that takes into account the correlation with neighboring blocks can find more accurate motions with relatively low computational complexity. Thus, notable image quality improvement was achieved using the proposed MCFI. For most of the test sequences, our proposed MCFI algorithm outperformed existing ones with respect to both the average PSNR and SSIM performances. In particular, the proposed MCFI provided PSNR and SSIM gains of up to 9.11 dB and 0.0996, respectively, for sequences containing multiple objects with complicated motions. Furthermore, our proposed MCFI reduced the computational complexity by up to 66.9 %.
